# Replicating Anatomical Teaching Specimens Using 3D Modeling Embedded Within a Multimodal e-Learning Course: Pre-Post Study Exploring the Impact on Medical Education During COVID-19

**DOI:** 10.2196/30533

**Published:** 2021-11-17

**Authors:** Chelsea Stunden, Sima Zakani, Avery Martin, Shreya Moodley, John Jacob

**Affiliations:** 1 Digital Lab BC Children's Hospital Vancouver, BC Canada; 2 Department of Pediatrics University of British Columbia Vancouver, BC Canada; 3 Division of Pediatric Cardiology University of British Columbia Vancouver, BC Canada

**Keywords:** congenital heart disease, cardiac anatomy, pathologic anatomy, education, learning aids, 3D models

## Abstract

**Background:**

The COVID-19 pandemic has had significant effects on anatomy education. During the pandemic, students have had no access to cadavers, which has been the principal method of learning anatomy. We created and tested a customized congenital heart disease e-learning course for medical students that contained interactive 3D models of anonymized pediatric congenital heart defects.

**Objective:**

The aim of this study is to assess whether a multimodal e-learning course contributed to learning outcomes in a cohort of first-year undergraduate medical students studying congenital heart diseases. The secondary aim is to assess student attitudes and experiences associated with multimodal e-learning.

**Methods:**

The pre-post study design involved 290 first-year undergraduate medical students. Recruitment was conducted by course instructors. Data were collected before and after using the course. The primary outcome was knowledge acquisition (test scores). The secondary outcomes included attitudes and experiences, time to complete the modules, and browser metadata.

**Results:**

A total of 141 students were included in the final analysis. Students’ knowledge significantly improved by an average of 44.6% (63/141) when using the course (SD 1.7%; Z=−10.287; *P*<.001). Most students (108/122, 88.3%) were highly motivated to learn with the course, and most (114/122, 93.5%) reported positive experiences with the course. There was a strong correlation between attitudes and experiences, which was statistically significant (*r*_s_=0.687; *P*<.001; n=122). No relationships were found between the change in test scores and attitudes (*P*=.70) or experiences (*P*=.47). Students most frequently completed the e-learning course with Chrome (109/141, 77.3%) and on Apple macOS (86/141, 61%) or Windows 10 (52/141, 36.9%). Most students (117/141, 83%) had devices with high-definition screens. Most students (83/141, 58.9%) completed the course in <3 hours.

**Conclusions:**

Multimodal e-learning could be a viable solution in improving learning outcomes and experiences for undergraduate medical students who do not have access to cadavers. Future research should focus on validating long-term learning outcomes.

## Introduction

### Background

Congenital heart disease (CHD) occurs in 1 out of every 100 births and is an essential subtopic of study for medical students [1]. One of the fundamental concepts in understanding the physiology of CHD is comprehending the anatomy of the normal heart and how that is different with congenital heart defects. An understanding of the gross anatomy lays the foundation for effective clinical tasks, such as performing physical examinations, evaluating medical images for diagnosis, and performing procedures required for intervention (eg, surgical correction).

### Anatomy Education

In the past, medical schools have used several creative approaches to introduce 3D visualization of complex structures into their curricula when cadavers were not available or feasible to use. Technologies discussed in the literature include computer-based learning (eg, videos, animations, multimedia simulation software, 3D models, and 3D computer graphics), 3D printed models, and extended realities (stereoscopic videos, virtual reality, and augmented reality). The tools are valuable teaching and learning aids [2-13] but are underused [14].

### Local Context

At our institution located in Canada, between 200 and 300 undergraduate medical students attend a 2-hour session where they use cadaveric specimens to work through the key features of common congenital cardiac abnormalities. Medical students enter this program with a completed undergraduate degree, then undergo 4 years of undergraduate medical studies, followed by a residency. It is widely accepted that cadaveric specimens are the gold standard for anatomy education, providing students with a 3D understanding of the human body, a sense of the relationship between different anatomical features, and an appreciation of the depth, fragility, and variability within the human body [2,15]. However, in pediatric medicine, there is a noteworthy difference with regard to access to cadaveric specimens because of various reasons, including improved outcomes within the affected patient population (9 of 10 patients with CHDs now live to adulthood [1]) and financial, religious, cultural, ethical, and legal considerations related to the acquisition and use of such specimens [16].

In combination with barriers that are well documented in the literature, we were also amid the COVID-19 pandemic, where a return to in-person cadaveric learning sessions was not permitted because of local public health and safety requirements [3,17]. Social distancing measures implemented in our area precluded students from gathering in group learning settings. Without alternatives to in-person cadaveric laboratories, the opportunity to use cadavers for anatomy learning was compromised for an indeterminate length of time. There was an urgent need for alternatives that allowed for 3D spatial visualization of complex structures under these circumstances.

### Study Objectives

With this in mind, we created a customized CHD e-learning course that contained interactive 3D models of anonymized pediatric congenital heart defects. The course was implemented for use in first-year undergraduate medicine courses. The aim of this study is to assess whether the e-learning course contributed to improved learning outcomes in a cohort of first-year undergraduate medical students. We hypothesized that students would gain new knowledge from the course. Secondary hypotheses were that students would report positive attitudes and experiences with the course and that student’s reported attitudes, experiences, and knowledge would be related, and experiences would be affected by the minimum requirements of individual hardware used to access the course.

## Methods

### CHD Cases Course Design

#### Pedagogy

The e-learning course was designed to incorporate pedagogical attributes along 5 parameters: (1) developing content, (2) storing and managing content, (3) packaging content, (4) student support, and (5) assessment [18]. The structure also addressed barriers to implementation identified by conceptual frameworks [19] such as technology compatibility, user design, student motivation, perceived usefulness and ease of use, and access.

This remote e-learning course was also well integrated with Fleming VARK model (visual, auditory, reading or writing, and kinesthetic learners) [20], providing course materials, which are tailored for different ways of learning. Visual learners are supported through the addition of videos, graphics, and animations. Auditory learners are supported through the addition of videos. Reading and writing learners are supported through didactic text within each module. Finally, kinesthetic learners are supported through the opportunity to interact with patient-specific 3D virtual models.

#### Developing the Course Content

**Figure 1:**
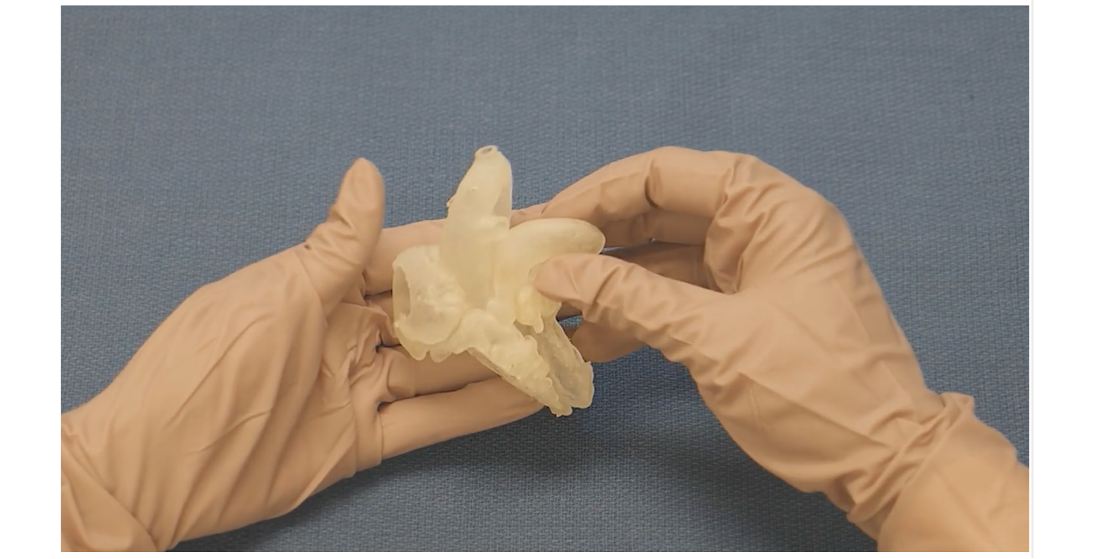
Video describing a heart with tetralogy of Fallot using a patient-specific 3D printed model.

**Figure 2:**
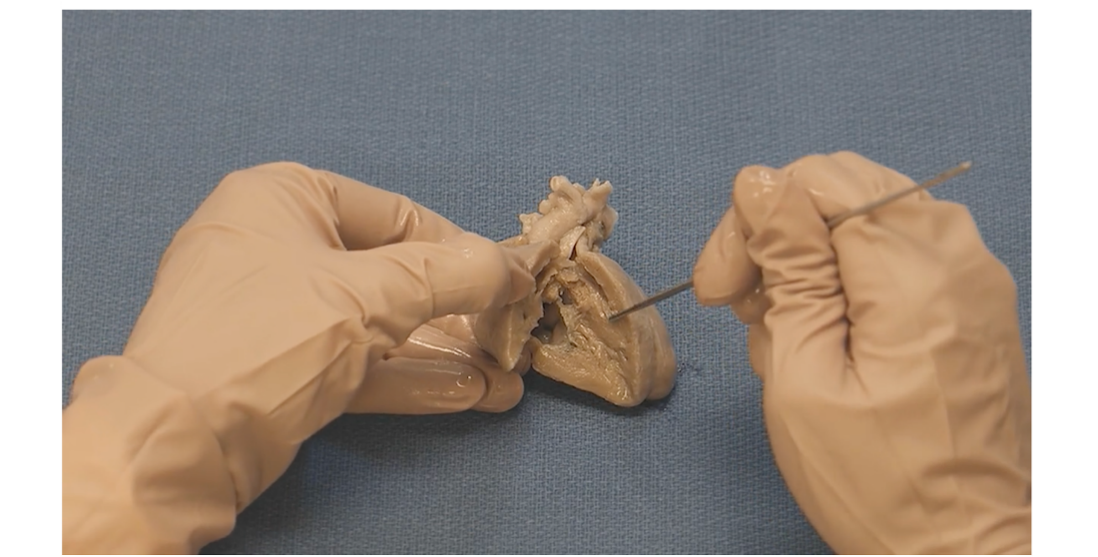
Video describing tetralogy of Fallot using a cadaveric specimen.

**Figure 3:**
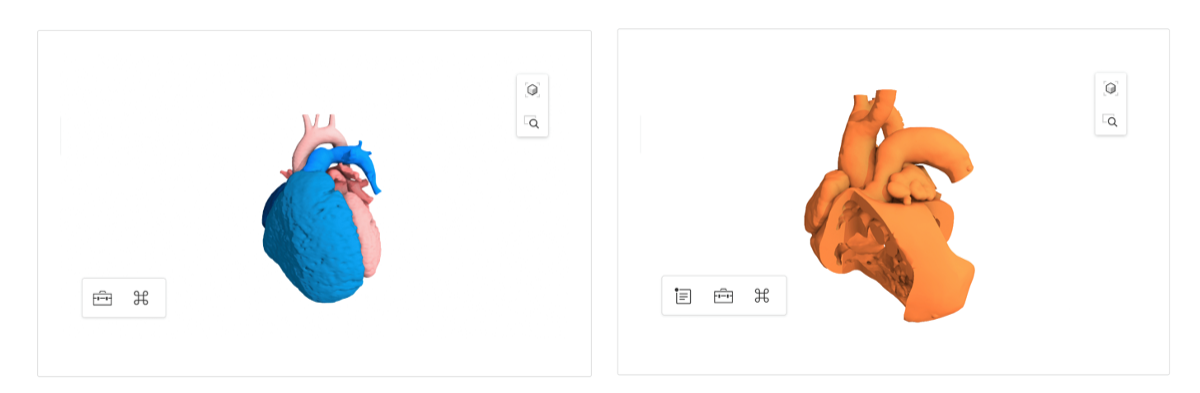
Interactive 3D virtual models of tetralogy of Fallot.

The CHD media was custom designed and developed by the BC Children’s Hospital Digital Lab. To ensure that the course was relevant, accurate, and aligned to the aims of the medical school curricula, e-learning objectives and course content were designed by a team of approximately 8 stakeholders, including cardiologists, researchers, medical students, and learning designers. Although increased collaboration requires more time, an interprofessional approach allowed us to combine the expertise of several fields for the development of a creative product. Media included videos of 3D printed models (Figure 1), cadaveric specimens (Figure 2), interactive 3D virtual models (disseminated via Modelo; Figure 3), animations, and graphics (acquired through Shutterstock). The interactive course content was supplemented with text and packaged as an e-learning course using Articulate Rise course authoring software. In addition, 1 faculty member (a pediatric cardiologist) provided overarching medical oversight and clinical quality assurance for the final course content.

Representative cadaveric learning cases with corresponding computed tomography scans of congenital heart defects were featured in the media of the course. It presented 6 CHD pathologies across a spectrum of disease severity: atrial septal defects, tetralogy of Fallot, transposition of the great arteries, coarctation of the aorta, truncus arteriosus, and hypoplastic left-heart syndrome. A *normal* heart was also represented for comparison.

#### Storing and Managing the Content

The e-learning course was made accessible via a direct link that could be posted to various learning management system platforms used by the teaching institution or disseminated via email; this mode of delivery was pragmatically selected instead of directly embedding the course within a learning management system to reduce potential for access issues during implementation, and to improve future reach [19].

Pre- and posttests were administered using Qualtrics, a survey data capture system, and seamlessly embedded within the course content. Students were made aware that the completion and the results of these quizzes were electronically tracked.

#### Packaging the Content

In this study, the e-learning course was offered to undergraduate medical students as a component of their studies on CHDs. The e-learning course was released after virtual lectures were hosted; however, student participation in these elements was not standardized, as it would not have been reflective of the current environment. Flexibility is a tenet of remote learning. The e-learning course was conceptualized as 11 modules, each consisting of an introduction, module-specific multimodal content, and learning checks (Textbox 1) [19].

**Modules of the e-learning course**

**The 11 modules of the e-learning course**

1. Course overview and learning objectives
2. Gross anatomical features of the normal heart
3. The sequential segmental approach
4. Atrial septal defects
5. Tetralogy of Fallot
6. Transposition of the great arteries
7. Coarctation of the aorta
8. Truncus arteriosus
9. Hypoplastic left-heart syndrome
10. Virtual reality models
11. Course summary

The content was presented in smaller manageable chunks (as individual modules) to allow for flexible learning and shareability. When new technologies (eg, virtual models) were introduced, a tutorial was available to provide orientation. The learning modules had to be completed sequentially, and each subsequent module was only accessible after passing a brief learning check on the prior module content. There were no explicit time constraints for the course, and completion was not mandatory, nor was it officially graded. Nonetheless, assessments (eg, pre- and postassessments) were presented as an optional part of the course, and students were made aware that the completion and the results of the quizzes were electronically tracked [19].

#### Student Support

During COVID-19, the status quo for anatomy sessions at our institution transitioned to virtual lectures over Zoom (Zoom Video Communications Inc) videoconferencing, in combination with web-based demonstrations and discussions. The e-learning course was added as an adjunct to these sessions to provide diverse teaching material for instructors [19].

#### Learning Check Assessments

*Learning check* assessments were created by an instructor and cardiac specialist and tested with students before being deployed within each module [19]. The goal was to reinforce the learning objectives through multiple-choice questions, true or false questions, matching questions, or short answer questions. To ensure the quality and validity of testing, the questions were developed by content experts and informed by the structure of current anatomy tests published in peer-reviewed literature [21,22]. Students had to receive at least 60% on each learning check to move onto the subsequent module. After completing the learning check, feedback was provided. Feedback included the student’s test score and referenced to the section that should be reviewed for incorrect responses.

### Principal Objective

The primary objective of this study is to determine whether undergraduate medical students gained new knowledge from the course. The secondary objective is to evaluate whether students reported positive attitudes and experiences from the course and whether any of the measures of attitude, knowledge, and experiences were related.

### Participants and Protocol

#### Protocol

A cohort of 290 first-year undergraduate medical students at a large postsecondary institution were selected for analysis because the cohort included a sufficiently powered number of students. Within the course, each student was invited to complete 3 optional questionnaires throughout the implementation (pretest, posttest, and feedback surveys). Questionnaires were embedded within the Articulate Rise e-learning course and hosted on Qualtrics to be completed remotely and concurrently with the course. On completion of the evaluation components, students were entered to a draw for 1 of 3 CAD $50 (US $40) Amazon Gift Cards. Ethics approval was granted by the Children’s and Women’s Research Ethics Board of the University of British Columbia (#H20-03660).

#### Inclusion and Exclusion Criteria

The inclusion criteria were that data had to be entered by first-year undergraduate medical students during November 2020. There were no exclusion criteria.

### Outcome Measurement

The primary outcome was learning outcomes. The secondary outcomes include attitudes and experiences with the e-learning modules, time to complete the modules, and browser metadata.

#### Learning Outcomes

Learning outcomes were measured as a factor of test scores (Multimedia Appendix 1). The mean of paired pretest and posttest scores was calculated and compared. Mean change in test scores was calculated and reported by subtracting pretest scores from posttest scores for each individual.

#### Attitudes

Attitudes were calculated as a factor of motivation to learn with the e-learning modules. Questions in the feedback pertained to motivation to learn and were included in the composite variable for attitudes. These questions were based on an adapted version of Zaharias and Poylymenakou’s usability survey [23].

#### Experiences

Experiences with the e-learning course were measured by Zaharias and Poylymenakou’s metrics on content and resources, media use, learning strategies design, self-assessment, and learning support [23]. Items that addressed experiences with the e-learning modules in the feedback were combined as a composite variable to integrate the multiple measurements into a single variable representing student experience with the learning module. Variables for experience included questions focusing on content and resources, media use, learning strategies design, self-assessment, and learning support.

#### Time to Complete

Time to complete the course was calculated using the time stamps in the metadata. Because the course was not administered on a learning management system, we used the time stamps from the pre- and posttests as a proxy measure of time. Total time to complete the course was calculated by subtracting the time stamp of the start of the posttest from the time stamp of the completion of the pretest.

#### Browser Metadata

Browser metadata were recorded by the e-learning module and included information about the browser, operating system, and screen resolution used by participants to access the e-learning materials. Metadata included information about the internet browser, operating system, and screen resolution. Browser metadata relationships were extracted from the course metadata and were compared with learning outcomes, attitudes, and experiences.

Internet browser information was acquired through the course metadata. As part of the technical requirements for the course, students were informed that the course did not function well in Internet Explorer and advised to use other web browsers to complete the course (eg, Chrome, Firefox, and Safari).

Operating system information was acquired through the course metadata. As part of the technical requirements for the course, students were advised that tablets, smartphones, and other mobile devices may not work in all areas of the course (eg, interactive 3D models), so they should use a PC or Mac-based computer to fully use the course materials.

Resolution is the number of pixels a screen can show, both horizontally and vertically, and it is generally agreed that higher-resolution devices provide a better experience when using immersive media. Students use a variation of hardware, and thus screen resolutions, to complete their school work. We included a measure of screen resolution to assess whether screen resolution impacted learning of the material and reported experiences. Screen resolution information was acquired through the course metadata.

#### Qualitative Feedback

The results of the open-ended user feedback were reviewed for relevant information related to attitudes and experiences.

### Analysis and Reporting

#### Power Calculation

A priori power analysis was calculated using G*Power 3 [24]. Assuming a small to moderate effect size (Cohen *d*z=0.35) with 95% power and probability of a type 1 error of .05, a total sample size of 109 was needed.

#### Analysis

Statistical analysis was performed using the IBM SPSS Statistics for Mac, Version 27 (IBM Corp.). Data were analyzed according to the protocol set; that is, students had to complete the pre- and posttests measuring learning outcomes to be included in the analysis.

Descriptive statistics were reported. To assess whether students’ learning outcomes improved with the e-learning course, test scores from the *pre and posttests* were analyzed using a Wilcoxon signed-rank test. Test scores were not normally distributed. We dichotomized scores such that no partial marks were provided for question responses (ie, all correct answers had to be selected for a *select all that apply* question). To investigate whether experience, attitudes, and test scores were related, a bootstrapped bias-corrected and accelerated Spearman rank-order correlation was performed. For missing values, a single value was filled for each missing value with the mean score for that question. The results of the feedback questionnaire were calculated using descriptive statistics and analyzed in SPSS statistical software.

The findings of open-ended feedback were coded using a rapid analysis framework [25]. This framework involves summarizing the comments in a table and then consolidating summaries on broad themes or categories with supportive quotes to indicate areas for improvement.

## Results

### Study Group

A total of 290 first-year undergraduate medical students enrolled in the course and were invited to use the e-learning modules as part of an educational series on congenital heart defects. Students engaged with the initial pretest 228 times, with the posttest 198 times, and the usability questionnaire 155 times. After obtaining consent and removing duplicate and incomplete responses, 141 students were included in the analysis.

### Learning Outcomes

**Figure 4:**
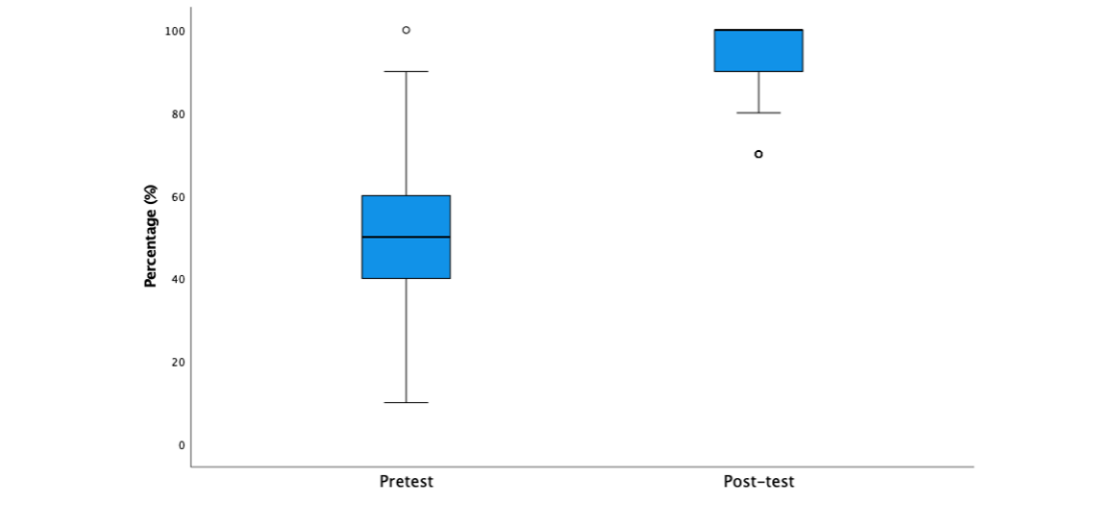
The median (middle quartile) test scores for the pre- and posttests taken by students using the e-learning course. Students’ knowledge significantly improved by an average of 44.6% between tests (SD 1.7%; Z=−10.287; *P*<.001). The circles denote outliers.

On the basis of the test scores measured before and after using the e-learning course, students’ knowledge significantly improved by an average of 44.6% between tests (SD 1.7%; Z=−10.287; *P*<.001; Figure 4). The median score for the pretest was 50.0% (IQR 20.0%, SD 15.7%), and the median score for the posttest was 100% (IQR 10.0%, SD 7.4%).

### Time to Complete

The time to complete the modules was calculated using the time stamps on the pretests captured before starting the course and from the posttests immediately following the completion of the course. The median time to complete the modules was 2.3 (IQR 4.7, SD 26.2) hours. Most students (83/144, 58.9%) completed the course in <3 hours. Some students completed the course within 1 day (47/144, 33.3%), and a few students completed the course within a few days (11/144, 7.8%). No relationships were found between the time and change in test scores (*P*=.75), attitudes (*P*=.20), or experiences (*P*=.17).

### Interactive Courses May Take More Time to Complete

Completion times for interactive modules is dependent on the time spent interacting with the modules and knowledge while entering the course. A small number of students reported that the course took longer than described in the course outline to complete. Of them, 1 student mentioned “To really take this module seriously it took longer than the 1.5 hours stated in the intro.”

### Attitudes

#### Overview of Attitudes

Regarding attitudes, 88.5% (108/122) of the students were highly motivated to learn with the course (Table 1). In total, 59.8% (73/122) of the students indicated that the course stimulated further inquiry with mentors or other students, 96.7% (118/122) indicated that the course was enjoyable and interesting, 94.3% (115/122) indicated that the course provided instruction or training that matched their experience, 97.5% (119/122) indicated that the course met their needs, and 93.4% (114/122) of the students indicated that the learning requirements and criteria for learning success were clear within the course.

We also asked questions about students’ motivation in the usability survey. Students mentioned that the course met their needs for learning congenital heart defects and that the course was of high quality.

**Table 1:** Students’ motivation to learn with the multimodal e-learning course (N=122).

	Strongly disagree	Somewhat disagree	Neither agree nor disagree	Somewhat agree	Strongly agree
The course stimulated further inquiry (eg, with mentors or other students), n (%)	2 (1.6)	4 (3.3)	43 (35.2)	36 (29.5)	37 (30.3)
The course was enjoyable and interesting, n (%)	2 (1.6)	0 (0)	2 (1.6)	36 (29.5)	82 (67.2)
The course provided instruction or training that matched my experience, n (%)	2 (1.6)	1 (0.8)	4 (3.3)	36 (29.5)	79 (64.8)
The course met my needs, n (%)	1 (0.8)	0 (0)	2 (1.6)	28 (23.0)	91 (75.0)
The learning requirements and criteria for learning success were clear within the course, n (%)	1 (0.8)	2 (1.6)	5 (4.1)	31 (25.4)	83 (68.0)

#### e-Learning Courses Can Meet Undergraduate Medical Student Needs for Learning Congenital Heart Defect Anatomy

Students reported that the course covered congenital heart defects in sufficient breadth and depth to meet the learning objectives. Students reported the following:

I was able to draw distinctions between different congenital heart diseases and differentiate between them.

I found this course to provide sufficient detail and information, allowing me to fill my knowledge gaps with respect to CHD.

However, when creating e-learning modules for undergraduate medical learning, students could benefit from a progress save function to allow for flexible learning experiences:

If [I] did not complete the module all in one go, I was bumped out and had to repeat all of the quizzes again before I could move forward. If there was a way to save your spot in the module, that would be very useful to be able to come back later!

Furthermore, when providing the module asynchronously, it is important to acknowledge how the pieces fit together. Some students were motivated to seek additional information:

It would be nice to provide success rates of surgeries/management.

Would love more information about the sequential analysis approach.

In general, students reported that “the [course] was so helpful.” However, administration may work better when offered in advance of live sessions, where students can ask questions to instructors and inquire about access to additional resources.

#### Innovative Multimodal Teaching Tools Can Help to Offer High-Quality e-Learning Experiences

Students were motivated by the multimodal web-based delivery afforded by the course. A student said:

I really enjoyed learning this way and I hope we can do this more often.

Students also described the content as of high quality, which influenced motivation to learn. The course offered a high-quality alternative to other learning experiences. A student commented:

Wow, this was an amazing module, probably one of the best online modules we’ve done so far. Best module I have encountered since entering med[icine].

### Experiences

#### Overview of Experiences

Regarding experiences, 93.5% (114/122) of students reported positive experiences with the course (Table 2).

**Table 2:** Students’ experiences with the multimodal e-learning course (N=122).

		Strongly disagree	Somewhat disagree	Neither agree nor disagree	Somewhat agree	Strongly agree
**Content and resources**						
	Concepts (ie, segmental approach, defects) were illustrated with concrete, specific examples, n (%)	0 (0)	4 (3.3)	1 (0.8)	30 (24.6)	87 (71.3)
	The vocabulary and terminology were used appropriately, n (%)	1 (0.8)	1 (0.8)	3 (2.5)	36 (29.5)	81 (66.4)
	The course covered congenital heart defects in sufficient depth to meet the learning objectives, n (%)	1 (0.8)	2 (1.6)	7 (5.7)	2 (1.6)	100 (82.0)
	The course was free from technical problems, n (%)	4 (3.3)	11 (9.0)	1 (0.8)	39 (32.0)	67 (54.9)
**Media use**						
	The text and images included had a strong connection to the learning objectives, n (%)	1 (0.8)	1 (0.8)	1 (0.8)	23 (18.9)	96 (78.7)
	Graphics and multimedia were used appropriately to assist in highlighting and learning critical concepts rather than merely entertaining or distracting me, n (%)	1 (0.8)	3 (2.5)	8 (6.6)	34 (27.9)	76 (62.3)
**Learning strategy design**						
	It was clear to me what was to be accomplished and what I would gain from using the course, n (%)	2 (1.6)	0 (0)	1 (0.8)	25 (20.5)	94 (77.0)
**Self-assessment**						
	Learning checks and other assessments adequately measured my accomplishment of the learning objectives, n (%)	1 (0.8)	0 (0)	0 (0)	28 (23.0)	93 (76.2)
**Learning and supports**						
	The course offered tools (resources, FAQs, glossary, and so on) that supported my learning, n (%)	1 (0.8)	4 (3.3)	18 (14.8)	45 (36.9)	53 (43.4)

#### Content and Resources

##### Experiences With Content and Resources

In terms of experiences with content and resources (Table 2), 95.9% (117/122) of the students reported that concepts were illustrated with concrete, specific examples; 95.9% (117/122) reported that the vocabulary and terminology were used appropriately, 91.8% (112/122) indicated that the course covered congenital heart defects in sufficient breadth and depth to meet the learning objectives, and 86.9% (106/122) completed the course with minimal technical problems.

##### e-Learning Courses Offer a Permanent Resource to Support Learning

We also asked questions about students’ experiences with the content and resources in the usability survey. Students mentioned that the course offered a permanent resource that supported their learning.

The e-learning course offered students a positive learning environment by offering resources and tools that were easily accessible. Students commented that the e-learning package “really helped to solidify the material.” However, providing students with a PDF export or key concept summaries to accompany modules may improve the note taking experience. Several students commented:

I would suggest creating some supplemental content by compiling images and notes from these modules into a PDF/PowerPoint slide so students have condensed take home messages to download and refer back to.

A final summary page would be nice of all the CHD and their key clinical presentations.

##### Technical Difficulties Can Create Inequities in Learning Experiences

Although most students did not describe having technical difficulties, we found the students who reported technical issues had difficulties interacting with the 3D virtual models, which prevented them from using them as instructed:

Myself and a few others in my class could not get the interactive models to load. Of the people who did get it to work, they reported it being slow and hard to use.

Our assessment included measuring some requirements for viewing the models, but we were not able to assess all the system requirements. Hardware, internet connections, graphics cards, and web browsers that do not meet the minimum requirements can impact experience and often explain why models are reported to be *slow*. The sample size for students using OS systems that did not meet the minimum requirements was small (1/141, 0.7%) and would not be large enough to detect differences in our analysis. This 1 student reported having significant technical difficulties.

#### Media Use

##### Experiences With Media Use

In terms of experiences with media use (Table 2), 97.5% (119/122) of students reported that the text and images included had a strong connection to the learning objectives, and 90.2% (110/122) reported that the graphics and multimedia were appropriately used to assist in highlighting and learning critical concepts rather than merely entertaining or distracting them.

##### Text and Images Improve Learning When There Is a Strong Connection to the Learning Objective

The course used images and text to highlight the key learning objectives of the course. The use of multiple types of media to convey concepts enhanced education quality. In addition, the use of patient-specific models increased value by offering real clinical examples. A student commented that they:

Very much liked how the blood flow was highlighted [in the blood flow animations], so that it was easy to understand what we would see clinically.

However, we did not duplicate all material across different modes of delivery (eg, text and videos), and some students mentioned that they missed this content. Providing video transcripts may help to alleviate this issue. A student commented as follows:

I also felt like some of the questions asked things that weren’t necessarily covered in the specimen videos. Overall, the specimen videos felt out of place at times compared to the text.

##### Graphics, Videos, and Multimedia Can Be Used to Highlight Learning Critical Concepts

The incorporation of graphics, videos, and 3D models offered students an interactive experience that allowed them to develop an understanding of the learning objectives through different ways of learning. In addition, the different modes of showcasing CHDs allowed the students to understand the concepts in different situations. Students commented as follows:

The videos of the real neonatal hearts were extremely helpful and well done, with a great level of detail.

The illustrations and videos were really helpful, especially for visualizing blood flow and the different procedures.

The interactive-ness and organization of the module is more effective way of learning.

In fact, students were seeking more diversity in all learning scenarios. A student commented as follows:

Would be nice to have static graphics showing blood flow in addition to animations.

However, the 3D virtual models that offered the students a more interactive form of learning were perceived by some students as more difficult to use. The interactive models focus on higher-order thinking skills, such as the ability to break the cardiac components down into their constituent parts to determine the relationship of the parts. These types of learning situations may require more support for new (first-year undergraduate) anatomy learners. The comment below highlights the sentiments offered by many students:

In the embedded models, it was difficult to see the defect discussed.

Interactive models for new learners could be improved by the addition of anatomical notations.

#### Learning Strategy Design

##### Experiences With the Learning Strategy Design

In terms of experiences with the learning strategy design (Table 2), 97.5% (119/122) of the students reported that it was clear to them what would be accomplished and what they would gain from using the course.

##### Learning Requirements Were Packaged for Learning Success

Students reported that the learning objectives within the course were clear:

I know what I’m supposed to be learning from this.

This course was easy to follow.

Furthermore, students found that the packaging of the content in small modules for flexible learning provided a suitable progression. Several students commented as follows:

I really enjoy going through things on my own when they are organized like this module.

[The course] provided us with lots of info but at the same time it was not overwhelming.

Another student said the following:

[I] learned a lot in a short amount of time, much more than having a 2-hour lecture on congenital heart disease.

#### Self-assessment

##### Experiences With the e-Learning Self-assessment

In terms of experiences with the e-learning self-assessment (Table 2), 99.2% (121/122) of the students indicated that the learning checks and other assessments adequately measured their accomplishment of the learning objectives.

##### Learning Checks Help to Measure Accomplishment of e-Learning Objectives

Our qualitative results suggested that the learning checks and other assessments adequately measured student accomplishment of the learning objectives. Students expressed appreciation for the learning checks, which allowed them to measure their learnings from the course. A student mentioned the following:

The knowledge checks really helped cement the material and know when I had to go back to review a concept.

However, students requested a larger question bank that offered greater diversification, so they could further test their understanding. Students wrote as follows:

I liked the integrated assessments, but it would be beneficial if there was a little more variety in questions.

#### Learning and Supports

##### Experiences With Learning and Supports

In terms of experiences with the learning and supports (Table 2), 81.1% (98/121) of the students indicated that the course offered tools that supported their learning.

##### The Segmental Approach and Congenital Heart Defects Can Be Taught on the Web

The multimodal components of the e-learning course were positively perceived by students for learning the segmental approach and congenital heart defects. The variety of videos, animations, text, images, and 3D modeling provided students with examples to solidify the concepts. Students commented as follows:

The animations of blood flow in a normal heart vs abnormal heart for each condition were very helpful when understanding blood flow (especially when color-coded to indicate where blood was mixing).

I really enjoyed the videos that helped me visualize the defects and the explanations that went along with it, walking me through the anatomy.

Furthermore, students preferred the course over traditional laboratories and lectures. Students reported as follows:

I learned so much more here than in traditional lectures. I hope we will have more of these learning modules in the future.

This module was more helpful than either of the lectures we have had so far on congenital heart defects.

I much prefer this method of learning via modules with graphical depictions, videos, and quizzes for our laboratory courses.

#### Relationship Between Attitudes, Experiences, and Test Scores

**Figure 5:**
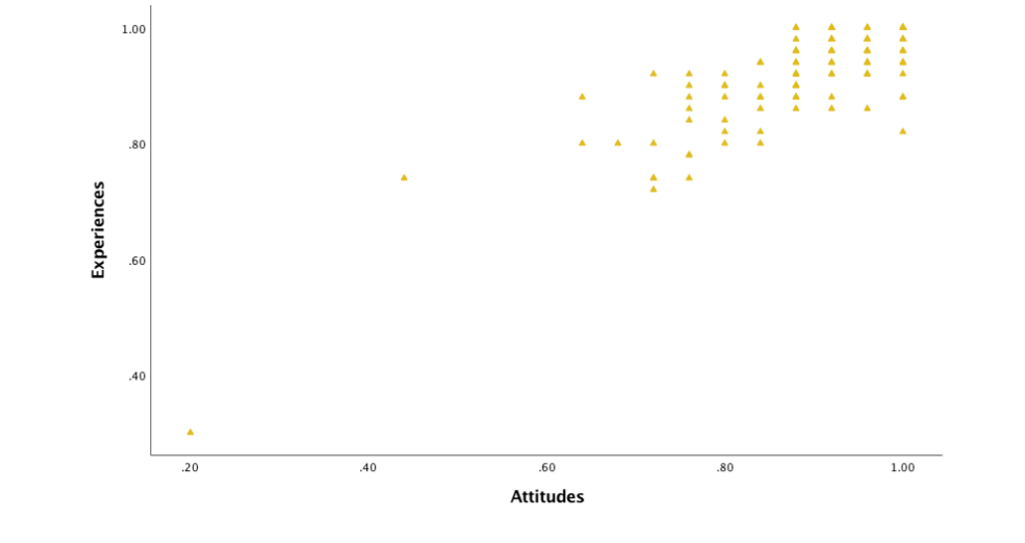
The relationship between student attitudes and experiences. There was a strong correlation, which was statistically significant (*r_s_*=0.687; *P*<.001; N=122).

To test for relationships between the students’ attitudes, experiences, and test scores, composite variables for attitudes and experiences were calculated and compared with the change in test scores. There was a strong correlation between attitudes and experiences, which was statistically significant (*r*_s_=0.687; *P*<.001; n=122; Figure 5). No relationships were found between change in test scores and attitudes (*P*=.71) or experiences (*P*=.47).

### Browser Metadata

Students used a variety of internet browsers, operating systems, and screen resolutions to access the course.

#### Internet Browser

Students most frequently completed the e-learning course with Chrome (Google: 109/141, 77.3%). Other browsers used to access the e-learning materials included Safari (Apple Inc: 17/141, 12.1%), Firefox (The Mozilla Foundation: 8/141, 5.7%), Edge (Microsoft: 5/141, 3.5%), and Chrome iPad (Apple Inc: 2/141, 1.4%).

#### Operating System

Students most frequently used Apple macOS (Apple Inc: 86/141, 61%) and Windows 10 (Microsoft: 52/141, 36.9%). Other operating systems used by students included iPads (Apple Inc: 2/141, 1.4%) and Windows 7 (Microsoft: 1/141, 0.7%).

#### Screen Resolution

Screen resolution of student devices ranged significantly. Ultrahigh-definition screens were considered above 1920×1080p, high-definition screens were considered between 1920×1080p and 1280×720p, and 720p was considered between 1280×720p to 852×576p. Most students had devices with high-definition screens (117/141, 83%), followed by ultrahigh definition (17/141, 12%), and 720*P* (7/141, 5%).

## Discussion

### Principal Findings

This study demonstrated that it may be possible to increase knowledge acquisition and positively impact student experiences and attitudes with multimodal e-learning courses when cadaveric specimens are rare, sparse, or otherwise impractical. This novel course followed successful implementation across 5 parameters: developing content with experts, storing and managing content in Rise Articulate, packaging multimodal content, offering student support through asynchronous e-learning, and confirming learning outcomes with assessments [18,26].

Our study responds to calls to support anatomy education using multimodal virtual approaches amid the COVID-19 pandemic [3,17,27,28]. The best methods for teaching anatomy are highly debated [2,15,29] because despite the availability of numerous teaching methods, students still report having insufficient anatomical knowledge [8]. Furthermore, the learning styles and associated learning needs of undergraduate medical students vary [30]. The variety of content and resources we presented were positively received by students, who reported that the concepts were illustrated with concrete, specific examples; that the vocabulary and terminology were used appropriately; and that the course covered congenital heart defects in sufficient breadth and depth to meet the learning objectives.

### Multimodal e-Learning and Knowledge Acquisition

Content and resources available within the course surrounded the study of 6 CHD pathologies across a spectrum of disease severity: atrial septal defects, tetralogy of Fallot, transposition of the great arteries, coarctation of the aorta, truncus arteriosus, and hypoplastic left-heart syndrome. A *normal* heart was also represented for comparison. Despite the range in complexity for each cardiac defect, knowledge acquisition was uniformly high across defects. In addition, students reported positive attitudes toward the course. This finding supports other investigations that have found digital anatomy courses to be a useful adjunct to teaching at the undergraduate level across a spectrum of disease severity [9].

In this study, the low learning outcomes before the course (pretest scores) followed by the uniformly high learning outcomes after using the e-learning course (posttest scores) suggest that a multimodal approach to addressing different ways of learning was effective in teaching first-year undergraduate students about varying degrees of CHD. Furthermore, students thought that the learning checks adequately measured their accomplishment of the learning objectives. These findings are consistent with other investigators who have found 3D visualization tools to be an effective method, particularly as adjuncts for improving learning outcomes compared with other methods of teaching anatomy [10-13,31]. Future implementations may want to consider the sequence of asynchronous implementation, as completion of e-learning before live sessions could yield better discussions. The approach would also benefit from studying how multimodal e-learning tools perform as an adjunct compared with cadaveric laboratories, especially in supporting longer-term learning throughout medical school and residency.

### Experiences and Attitudes With Multimodal e-Learning Materials

Our course translated high-resolution medical images into various novel learning tools (3D printed models and interactive virtual models) that were supplemented with simple materials (illustrations, animations, text, and teaching videos). Virtual learning environments that are low cost, offer self-assessments, are easy to use, align with the curriculum, have good graphics, and use simple material (such as plastic models and illustrations) are most sought by medical students [32,33]. Students in this study reported positive experiences with the multimedia; this suggests that some students may even prefer these modes of delivery. These findings support other investigators who have found that e-learning can improve access to learning materials, and that students are often highly satisfied with them [10-13,17,34,35].

Even though the course integrated many types of media that can have barriers during implementation [19], most students in this study reported that the end product had minimal technical problems and that the course offered tools that supported their learning. This is likely a factor of students using appropriate browsers, screen resolutions, and devices that supported the technical requirements of the course. We found that 1 student used hardware that did not meet the minimum requirements of the interactive virtual models and had resulting technical difficulties. For the other small percentage of students who reported technical difficulties, it was not clear from the results whether this was a factor related to access to the minimum hardware requirements or students not reading the technical requirements of the course. These issues should be closely monitored during implementation to ensure students have equitable learning experiences.

This study does highlight a concern associated with engaging students on the web, which is documented elsewhere [19]. Most students in this course indicated that they felt neutral about the course stimulating further inquiry with mentors or other students. An absence of social interaction between e-learners and instructors may emphasize a sense of disconnect in comparison with the traditional face-to-face learning environment [19]; it is hypothesized that this was also exacerbated by the COVID-19 pandemic. Recent editorials have expressed concerns about the transition to home-based learning and the struggle with establishing boundaries between work and home, which could affect faculty, students, and support staff [36].

### Development of Multimodal e-Learning Materials

The development of this course involved an interprofessional team that included several professionals and students who learned from and about each other to improve collaboration and the quality of the product. Previous research shows that interprofessional teams can reduce the risk of duplication and fragmentation and reduce costs associated with delivery [37-39]. We echo other investigators who suggest that interprofessional teams involving students bring an important and unique experience to course development [39,40]. We included residents and medical students during this e-learning course development because as the end users, students have a deep understanding of what they need to feel prepared for the workplace. However, we did experience some challenges associated with the number and composition of stakeholders, their different goals, interests, mental models, and agreement on the outcomes and structure of the course. These challenges have been previously reported by other teams working in interprofessional teams [39]. We echo other investigators in their sentiments around expecting a lot of work, accepting resistance, and communicating and listening frequently when engaging in interprofessional product development [40].

### Limitations

This study describes a group studied over a single point in time after the e-learning course was introduced during the COVID-19 pandemic. Changes in learning outcomes and attitudes and experiences are presumed to be the result of the e-learning course, but no control or treatment group was used. As such, it is not possible to dismiss other hypotheses or explanations for improved knowledge and positive attitudes and experiences. In addition, the longer-term retention of the acquired knowledge is unknown.

This study is also subject to several biases. The study is subject to information and selection bias, as we recruited participants through a first-year undergraduate medical course and offered a draw for a gift card. Motivation and reported outcomes related to using the course could have been impacted by these extrinsic motivations (eg, the draw). In addition, this study is subject to response bias because learners answered questions before the course, engaged in the e-learning modules, and then answered the same questions again after finishing the course.

### Conclusions

Access to alternative and adjunct options to cadaveric learning is beneficial and offers potential solutions to accessibility, economic, and ethical limitations to the current standard. Students may improve their understanding of medical materials, have a greater overall learning experience, and have greater motivation to learn the course contents. Future studies should assess long-term knowledge retention, compare multimodal e-learning to cadaveric laboratories, and include ways for interacting with other learners and mentors to reduce isolation.

## Appendix

Multimedia Appendix 1 Pre- and posttest questions administered to first-year medical students to assess learning outcomes.

## Abbreviations

CHD: congenital heart disease
