# Supplementary material for: Replicating Anatomical Teaching Specimens Using 3D Modeling Embedded Within a Multimodal e-Learning Course: Pre-Post Study Exploring the Impact on Medical Education During COVID-19
Source: JMIR Med Educ. 2021 Nov 17;7(4):e30533. doi: 10.2196/30533 (PMC8663546; doi:10.2196/30533)
Supplement: Multimedia Appendix 1 [file mededu_v7i4e30533_app1.docx]

MEDD411 - Pre-Test

What are the four chambers of the heart? (select all that apply)

- Ventricles (LV and RV) (19)
- Superior and Inferior Vena Cava (20)
- Atria (LA and RA) (21)
- Aorta (22)
- Pulmonary Artery (23)
- Patent Ductus Arteriosus (24)

What is the difference between the right and left atrium?

- The right atrium is characterized by certain features including the broad based triangular shaped appendage with trabeculations that extend beyond the atrium. (1)
- The left atrium is characterized by certain features including the broad based triangular shaped appendage with trabeculations that extend beyond the atrium. (2)
- The right atrium gives rise to an outflow tract with a sleeve of tissue called the infundibulum (3)
- The left atrium gives rise to an outflow tract with a sleeve of tissue called the infundibulum (4)

Which chamber gives rise to an infundibulum?

- Right atrium (1)
- Left atrium (2)
- Right ventricle (3)
- Left ventricle (4)

What are the methods to close a hemodynamically significant secundum atrial septal defect? (select all that apply)

- Surgical closure using a patch (1)
- Interventional catheterization using a device (2)
- An atrial switch operation (4)
- None of the above (3)

What is the 'tetralogy' referred to in tetralogy of Fallot? (select all that apply)

- Ventricular septal defect (1)
- Overriding aorta (3)
- Pulmonary stenosis (6)
- Right ventricular hypertrophy (10)
- Atrial septal defect (9)

True or False:<div>Tetralogy of Fallot is considered a type of cyanotic heart disease.</div>

- True (1)
- False (2)

How is transposition of the great arteries fixed?<span style="font-size:13px;"> (select all that apply)</span>

- A balloon atrial septostomy (1)
- An arterial switch operation (2)
- The Mustard (3)
- None of the above (4)

What clinical clues suggest coarctation of the aorta? (select all that apply)

- The pulses in the legs (femoral pulses) are reduced. (2)
- The pulses in the legs (femoral pulses) are elevated. (5)
- The blood pressure in the arms will be elevated. (3)
- The blood pressure in the arms will be reduced. (6)
- There is no effect on pulse or blood pressure. (1)

What is the definition of truncus arteriosus?

- A hole or defect in the septum or wall between the two atria. (2)
- A critically underdeveloped left side of the heart, referred to as "single ventricle anatomy". (3)
- A left sided obstructive lesion characterized by a narrowing of the candy cane shaped great vessel at the juxtaductal area that can present at different times in life. It is often associated with other left sided pathology. (4)
- A common type of cyanotic congenital heart disease characterized by the circulation being connected in parallel rather than series. (5)
- A lesion characterized by a single arterial vessel that gives rise to the systemic, pulmonary and coronary circulations. (1)

What medication can be lifesaving in a newborn baby with hypoplastic left heart syndrome?

- NSAIDs (1)
- Beta-blocker (2)
- Prostaglandin E1 (4)

End of Block: Pre-Test
